# Economic autonomy as a determinant of physical activity behavior in Chinese older adults

**DOI:** 10.3389/fpubh.2024.1466710

**Published:** 2025-02-20

**Authors:** Yuanheng Liu, Xianglin Luo, Hao Xu

**Affiliations:** ^1^Hunan Vocational College of Electronic and Technology, Changsha, Hunan, China; ^2^College of Physical Education, Hunan Normal University, Changde, Hunan, China; ^3^Hunan Applied Technology University Changde, Changde, Hunan, China

**Keywords:** the old people, physical activity, economic autonomy, China, impact study

## Abstract

**Background:**

The physical activity of the old people is affected by many factors, and the economic situation is an important factor affecting the physical activity. However, the relationship between economic autonomy and physical activity patterns among older adult Chinese has not been fully studied.

**Objective:**

To investigate the association between different types of economic autonomy and physical activity patterns among Chinese older adults aged 60 and above.

**Methods:**

Cross-sectional analysis of 1,961 participants from the 2018 China Health and Retirement Longitudinal Study (CHARLS). Economic autonomy was categorized into autonomous and non-autonomous groups. Physical activity was assessed through type, frequency, duration, and purpose, using validated questionnaires.

**Results:**

Economic autonomy showed positive associations with low and moderate-intensity physical activities (*p* < 0.05). However, economically non-autonomous individuals demonstrated higher participation in high-intensity physical activities, primarily due to work-related demands (78.7%). The frequency of physical activity was significantly different among economic autonomy groups (*p* < 0.01).

## 1 Introduction

Aging is an important issue in the development of the world, according to the projections made by the United Nations, the proportion of people over 65 years old in the world will rise from 10% today to 16% by 2050 (1). The same trend is evident in China’s aging population (2). The large aging population base will have a significant impact on the development of the world. In order to actively promote the development of the aging population, the World Health Organization proposed in 2021–2030 to promote the healthy aging of older adult people, which is defined as the development and maintenance of functional abilities that enable older people to be healthy (3). The realization of healthy aging is closely related to the healthy lifestyle of the older adult and the effective public health policies, and physical activity has always been regarded as an important element of a healthy lifestyle. In the existing research, physical activity has been proven to improve the mental health of the older adult and has significant meaning in preventing depression (4, 5). Moderate and high-intensity physical activity can effectively reduce the muscle loss associated with aging and improve the physiological level of the old people (6). Physical activity can also reduce the mortality rate related to Alzheimer’s disease and improve the life expectancy of the old people (7). It can also reduce the incidence of fatal falls that threaten the lives of the older adult and reduce the chance of falling in old people (8). It can also reduce the incidence of chronic diseases in the older adult and improve their quality of life (9). It can improve the physical function of the old people and slow down aging (10). The significance of physical activity in promoting the healthy aging of the older adult is self-evident, but the insufficiency of physical activity among the older adult is also a common problem in the world (11). The lack of physical activity among the older adult is prone to form a series of chronic diseases and squeeze the economy (12). It affects the physical and mental health of the older adult and the development of society. How to promote the physical activity of the older adult to a greater extent is a topic of common concern in society. The World Health Organization has formulated the “Global Action Plan for Physical Activity 2018–2030” to promote the level of physical activity (13). Scholars all over the world are also studying how to better promote the physical activity level of the older adult. There are many influencing factors for the participation of the older adult in physical activity. Guo Huijie found that factors such as public sports service, digital economy development level, marital status and education level have positive promoting effects on the physical activity level of the older adult in China (14). JeanZhang found that self-efficacy, social support and motivation had a positive impact on the physical activity of British older adult people (15). Among the many factors that affect the physical activity of the older adult, the economic situation of the older adult has always been an important factor that cannot be ignored. In the current studies in developed countries, the economic situation of the older adult has a strong correlation with the level of physical activity (16, 17). Based on this, scholars in developed countries have made a large number of studies to promote the development of physical activity of the older adult (16–20), and scholars in different countries have intervened in physical activity of the older adult from different angles (10, 21–23). The better the economic condition of the older adult, the higher the level of physical activity. Scholars have noted that, unlike in Western societies, the Chinese family holds unique significance in the process of social development. In China, the family plays a crucial role in both the upbringing of children and the care of the older adult (24). Parents will provide substantial economic support for their children’s education, marriage, and employment, and in return, children will provide economic support for their parents’ later life on a regular basis (25), a family system that is different from that of the West. In addition to the mutual assistance among family members, the government has established the “low-income protection” and “five-guarantee” projects to provide relief to groups in need of economic assistance (26), but the economic support provided is limited. The majority of the older adult living in China are farmers, and as they age, they gradually retire from their jobs without having sufficient pension income. The economic situation of Chinese older adult people is completely different from that of Western older adult people, as many older adult people still need to engage in labor after entering the older adult stage. What are the unique features in the physical activity of these older adults in China with different economic situations, and what should interventions look like? Given this, the article defines the study of “economic autonomy” to describe the economic situation of older adults, and conducts research on the relationship between economic autonomy and physical activity in order to dialogue with the physical activity theory of developed countries. Starting from this perspective is conducive to breaking through the limitations of Western-centric ideology, discovering the influencing factors of physical activity for older adult people in China similar to developing countries, and filling the current theoretical gap. It provides reference for countries with similar economic situations in China and thus affects the public health policies of other countries, thereby improving the physical health level of older adult people globally. The article will use the data from the 2018 CHARLS follow-up survey to investigate the relationship between economic autonomy and physical activity patterns of adults aged 60 and above in China. Specifically, the goals are as follows: 1. To analyze the relationship between different types of economic autonomy and physical activity levels. 2. To study how economic autonomy affects the types, frequency, duration, and purpose of older adult people’s sports activities.

## 2 Methods

### 2.1 Research design

Using a cross-sectional study based on data from the 2018 China Health and Retirement Tracking Survey (CHARLS), which covers 450 villages/urban communities in 150 countries/regions across the country, involving 17,708 people in 10,257 households on a 2-year cycle, the data used in this study was collected from 2018. This study included 1,961 sample sizes, including the economic and physical activity of 1,961 older adult people. We divided the economic situation into two types: economic autonomy and economic non-autonomy, in which economic non-autonomy was further divided into family social co-dependence type and complete social dependence type, and physical activity included low, medium and high intensity.

In the investigation, the investigators adopted effective quality control measures, including quality control training for interviewers. In order to ensure data quality, the regulator still adopts conventional quality control measures, such as the use of data verification and audio callback computer aided access system (CAPI), which greatly facilitates the supervisor to identify and correct the irregular access behavior of interviewers in a timely manner. When the interviewer is still visiting the village, the specially developed quality control system will immediately inform the interviewer of irregular visit behavior (such as irregular jump questions, incomplete questions or too fast questions).

Other verification measures include: comparing photos of interviewees from different rounds to ensure they are the same person; Check the recording; Conduct a brief telephone return interview with respondents. The CAPI system allows teams to send data back to headquarters via the Internet at the end of each day for timely checks. All interviewers’ first interviews will be checked. For visitors with relatively low verification scores, the monitors checked their follow-up visits more frequently. Normally, the visitor will receive feedback from the quality control team within 48 h of the end of the visit. This ensures that some mistakes are corrected before the team moves on to the next village.

In order to maintain the consistency of the sample, the missing value and the wrong value are eliminated.

Statistical significance was limited by P less than 0.01.

### 2.2 Participant characteristics

In the selection of samples, we only use the data of people over 60 years old, and we screen people according to age.

### 2.3 Procedural documentation

The economic autonomy of a living unit is determined by the income and expenses of its members, which are primarily collected through Questionnaire (27). These questionnaires encompass wage income, pensions, agricultural income, livestock and aquatic product income, self-employment income, and business income and expenses. When the economic income of a living unit exceeds its economic expenses, it is classified as economically autonomous. Conversely, when the economic income is less than the expenses, it is classified as economically non-autonomous.

### 2.4 Statistical analysis

This paper uses Spss25 for empirical analysis. Statistical analysis, regression analysis and adjustment effect analysis were used. With *p* < 0.1 as the significance level, the reliability of the research results is ensured within a certain confidence interval.

In the stage of statistical analysis, descriptive statistics are used to comprehensively understand the characteristics of the data, such as the central tendency and the degree of dispersion, and calculate the mean value and standard difference of each variable.

In the regression analysis, a suitable hierarchical regression model is constructed according to the theory and research hypothesis, and the dependent variables and independent variables are included. Spss25 was used to estimate the parameters of the model, and the key indexes such as regression coefficient, standard error and T-value were obtained. According to the regression results, the direction and degree of influence of independent variables on dependent variables were analyzed, and the significant independent variables (*p* < 0.1) were focused on and deeply interpreted.

On the basis of the main regression model, we introduce the regulating variables and the interaction terms between the independent variables and the regulating variables. Spss25 was used again to estimate the model parameters, paying special attention to the coefficients of the interaction terms and their significance. If the interaction term is significant at the *p* < 0.1 level, the moderating effect is present.

### 2.5 Old people

The database contains data on middle-aged and old people individuals over 45 years old, but the focus of this study is specifically on the old people population. Since the World Health Organization defines individuals over 60 years old as old people, the re-searcher has decided to exclude individuals under 60 years old from the analysis based on the research requirements.

### 2.6 Physical activity

The physical activity levels of the old people were collected through questionnaire (27), which mainly addressed the types of exercise, frequency, duration, and purpose of exercise. The relationship between economic autonomy and physical activity was deter-mined based on the number of individuals engaged in physical activity. The types of physical activity recorded only included those that lasted for more than 10 min per session. These types encompassed high-intensity physical activity (activities that make you breathe very hard), moderate-intensity physical activity (activities that make you breathe somewhat harder than normal), and low-intensity physical activity (such as walking).

The calculation of exercise frequency only considered the number of sessions per week that involved physical activity lasting for more than 10 min. The duration of exercise was categorized into four ranges: 10–30 min, 30 min to 2 h, 2–4 h, and more than 4 h. The purposes of physical activity included work, recreation, physical exercise, and others.

### 2.7 Economic autonomy

The economic autonomy of a living unit is determined by the income and expenses of its members, which are primarily collected through Questionnaire (19). These questionnaires encompass wage income, pensions, agricultural income, livestock and aquatic product income, self-employment income, and business income and expenses. When the economic income of a living unit exceeds its economic expenses, it is classified as economically autonomous. Conversely, when the economic income is less than the expenses, it is classified as economically non-autonomous.

The calculation of a living unit’s income only includes the primary members (husband and wife) or individuals without a marital status. Sources of income encompass wage income, pensions, agricultural income, livestock and aquatic product income, as well as self-employment and business income. Expenses cover all outlays such as rent, food, clothing, and more Families with living unit income exceeding expenses are termed economically autonomous families, while those with living unit income falling short of expenses are classified as economically non-autonomous.

## 3 Results

### 3.1 Sample characteristics

As shown in the Table 1, a total of 1961 participants were included in this study, older adults with a mean age of 69.51 years (standard deviation 7.05). In terms of gender, there were slightly more women participants than men, 1,013 (51.7%) and 948 (48.3%) respectively. In terms of educational attainment, the level of education of the participants was generally low, with 598 (30.5%) illiterate, 845 (43.1%) having only primary education, and only 518 (26.4%) having secondary education or above. In terms of living conditions, the majority of the older adult live without their spouse (1,485 persons (75.7%)), and 476 persons live with their spouse (24.3%). In addition, in terms of economic autonomy, 1,219 seniors (62.2 percent) had no financial autonomy, while 742 seniors (37.8 percent) had financial autonomy.

**Table 1:** Sample characteristics.

Variable		Frequency/mean	Economic autonomy	
			No	Yes
Age		69.51 (7.05)	69.58 (7.07)	69.39 (7.01)
Gender	Female	1,013 (51.7%)	643 (32.79%)	370 (18.87%)
	Male	948 (48.3%)	576 (29.37%)	372 (18.97%)
Education level	Illiteracy	598 (30.5%)	403 (20.55%)	195 (9.94%)
	Elementary school	845 (43.1%)	551 (28.10%)	294 (14.99%)
	Secondary school and above	518 (26.4%)	265 (13.51%)	253 (12.90%)
Living together	No	1,485 (75.7)	103 (5.25%)	0 (0.00%)
	Yes	476 (24.3%)	1,116 (56.91%)	742 (37.84%)

### 3.2 Physical activity patterns

As shown in the Table 2 Physical activity can be divided into high intensity physical activity, medium intensity physical activity and low intensity physical activity. In general, Chinese older adult people prefer to engage in moderate intensity physical activity and low intensity physical activity. The group engaged in high intensity accounted for 26.5%, the older adult engaged in moderate intensity accounted for 45.1%, and the group engaged in low intensity accounted for 81.9%.

**Table 2:** Physical activity intensity.

		Frequency	Percentage	Effective percentage
H	No	1,440	73.4	73.5
	Yes	520	26.5	26.5
	Total	1960	99.9	100.0
M	No	1,076	54.9	54.9
	Yes	884	45.1	45.1
	Total	1960	100.0	100.0
L	No	355	18.1	18.1
	Yes	1,605	81.8	81.9
	Total	1961	100.0	100.0

As shown in the Tables 3–5 In terms of the purpose of physical activity participation, 78.7% of the high-intensity physical activities were for the purpose of work demand; The purpose of physical exercise accounted for 14%, and entertainment accounted for 1.7%; Others accounted for 5.6%. The purpose of medium intensity work demand accounted for 50.5%; The purpose of physical exercise accounted for 18.9%; Entertainment purposes accounted for 2.8%; Other purposes accounted for 27.8%. The purpose of work demand in low intensity physical activity accounted for 34.3%; the purpose of physical exercise accounted for 43.5%; Entertainment demand accounted for 9.5%; Others accounted for 12.7%. This indicates that a considerable number of older adult people in China are still working after the statutory retirement age, and the payment of pensions for the older adult in China mainly depends on the government, but the Chinese government has not established a complete pension security system for the older adult, which makes the older adult still face life pressure after retirement, and occupies the time to engage in sports.

**Table 3:** High intensity physical activity purpose.

		Frequency	Percentage	Valid Percentage	Cumulative Percentage
Valid	Work	409	20.9	78.7	78.7
	Entertainment	9	0.5	1.7	80.4
	Physical exercise	73	3.7	14.0	94.4
	Others	29	1.5	5.6	100.0
	Total	520	26.5	100.0	
Missing	System	1,441	73.5		
Total		1961	100.0		

**Table 4:** Moderate intensity physical activity purpose.

		Frequency	Percentage	Valid percentage	Cumulative percentage
Valid	Work	446	22.7	50.5	50.5
	Entertainment	25	1.3	2.8	53.3
	Physical exercise	167	8.5	18.9	72.2
	Others	246	12.5	27.8	100.0
	Total	884	45.1	100.0	
Missing	System	1,077	54.9		
Total		1961	100.0		

**Table 5:** Low intensity physical activity purpose.

		Frequency	Percentage	Valid percentage	Cumulative percentage
Valid	Work	551	28.1	34.3	34.3
	Entertainment	152	7.8	9.5	43.8
	Physical exercise	698	35.6	43.5	87.3
	Others	204	10.4	12.7	100.0
	Total	1,605	81.8	100.0	
Missing	System	356	18.2		
Total		1961	100.0		

In terms of the frequency of physical activity participation, 56% of the groups engaged in three types of physical activities per week are engaged in more than 3–5 times, 38.9% of the groups are engaged in more than 5 times, and the groups engaged in 0–2 times per week only account for 5.1% of the total number, indicating that most Chinese older adult people have a stable physical activity habit at present.

In terms of the duration of physical activity, the frequency of physical activity in the older adult was the highest in the 30–2 h and 2–4 h periods, of which 30–2 h accounted for 47.3% and 2–4 h accounted for 35.9%. More than 4 h accounted for 10.8%, less than 30 min accounted for 6%.

### 3.3 Economic autonomy and physical activity association

As shown in the Tables 6–8 that economic autonomy affects the participation of Chinese older adult people in physical activity. The group of economically independent older adult people is positively correlated with low and moderate intensity physical activity (*p* < 0.05). The more economically independent older adult people are, the more physical activity they have in the middle and low intensity categories. After the inclusion of demographic characteristics, economic autonomy still has a significant positive impact on physical activity, while age and education level also have a significant impact, that is, the higher the age, the lower the weekly exercise frequency, and the higher the education level, the higher the weekly exercise frequency. However, the economically non-autonomous group showed higher participation in high-intensity physical activity, largely due to work-related demands (78.7%). This is also demonstrated by the regulating effect of physical activity for work purposes, as shown in the table. When the higher economic autonomy brings high-intensity work, the motivation to work will be reduced. Therefore, there were significant differences in the frequency of physical activity between economically self-governing groups (*p* < 0.01).

**Table 6:** Economic autonomy and physical activity.

		Economic autonomy		Chi-squared test	p
		No	Yes		
Engage in high-intensity exercise for 10 minutes per week	No	886	554	1.024	0.312
	Yes	333	187		
Engage in moderate-intensity exercise for 10 minutes per week	No	697	379	6.77	0.009
	Yes	522	362		
Engage in low-intensity exercise for 10 minutes per week	No	236	119	3.385	0.066
	Yes	983	622		
Days of high-intensity exercise for 10 minutes per week	0–2	62	38	0.165	0.921
	3–5	968	594		
	6–7	189	110		
Days of moderate-intensity exercise for 10 minutes per week	0–2	81	56	7.927	0.019
	3–5	798	439		
	6–7	340	247		
Days of low-intensity exercise for 10 minutes per week	0–2	42	23	3.972	0.137
	3–5	326	170		
	6–7	851	549		
	≥4 Hours	133	84		

The shaded part of yellow indicates economic autonomy and correlation with that activity.

**Table 7:** Economic autonomy and physical activity.

Model		Unstandardized coefficients		Standardized coefficients	*t*	Sig.
		B	Std. Error	Beta		
1	(Constant)	5.719	0.069		82.458	0.000
	Economic autonomy or not	0.271	0.113	0.054	2.404	0.016
2	(Constant)	9.372	0.628		14.912	0.000
	Economic autonomy or not	0.207	0.111	0.041	1.862	0.063
	Gender	0.053	0.115	0.0011	0.457	0.648
	Age	−0.060	0.008	−0.176	−7.510	0.000
	Educational level	0.243	0.077	0.076	3.141	0.002
	Living Together	0.088	0.131	0.016	0.672	0.501

**Table 8:** Moderated mediation effect.

		Economic autonomy	work	Economic autonomy * work	$R^{2}$
High-intensity	Model 1	0.105*** (3.011)			0.018
	Model 2	0.074** (2.181)	−0.279*** (−6.330)		0.093
	Model 3	0.204** (2.569)	−0.201*** (−3.264)	−0.159* (−1.808)	0.099
Moderate-intensity	Model 1	0.029 (0.743)			0.001
	Model 2	0.026 (0.685)	−0.243*** (−6.039)		0.055
	Model 3	0.057 (1.065)	−0.241*** (−5.953)	−0.014 (−0.82)	0.056
Low-intensity	Model 1	−0.013 (−0.475)			0.001
	Model 2	−0.017 (−0.642)	0.248*** (9.547)		0.061
	Model 3	−0.016 (−0.616)	0.248*** (9.536)	0.001 (−0.037)	0.061

*Represents a significant level of 0.1, **represents a significant level of 0.05, and ***represents a significant level of 0.01.

### 3.4 Physical activity participation model

**Figure 1:**
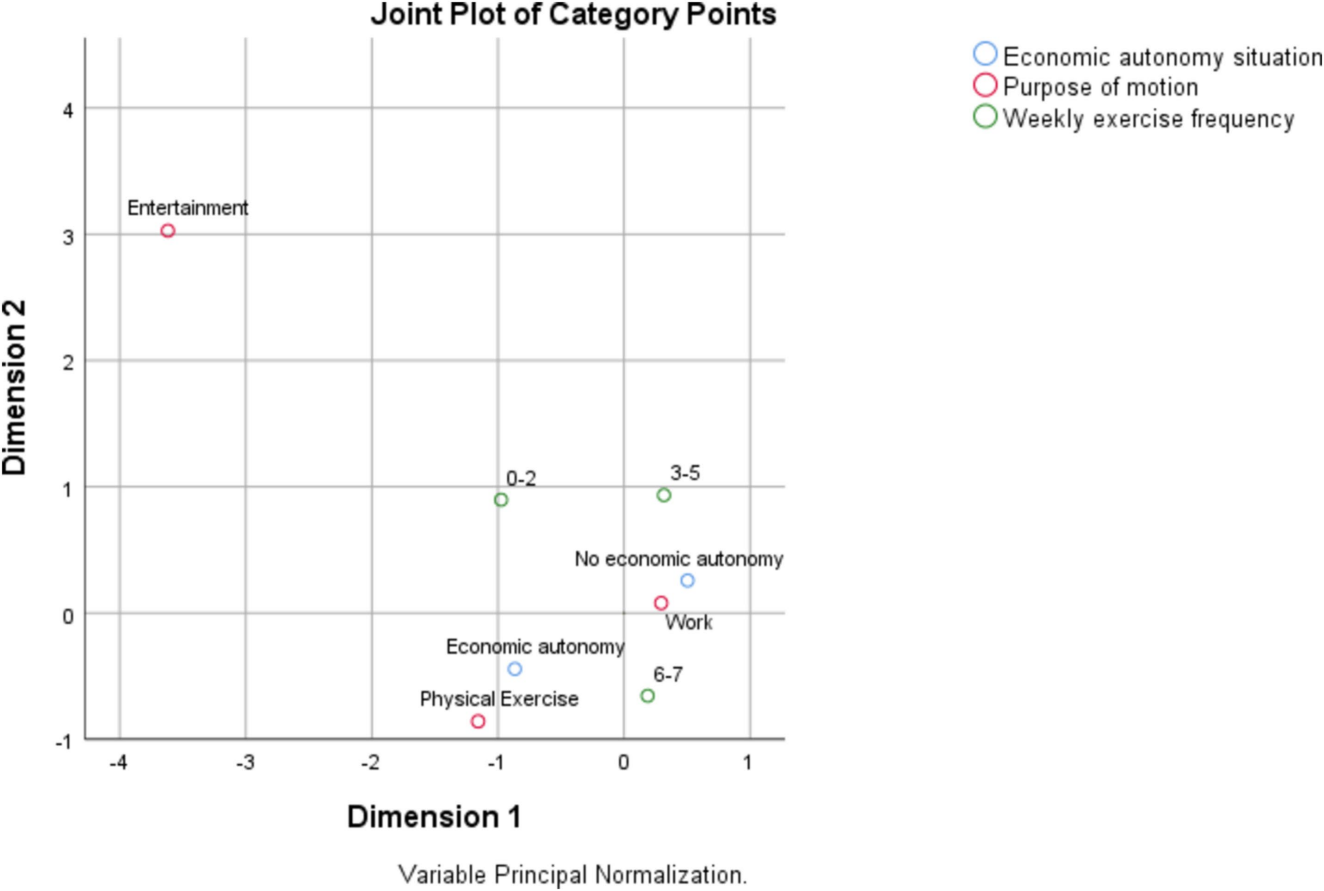
High intensity physical activity participation model.

**Figure 2:**
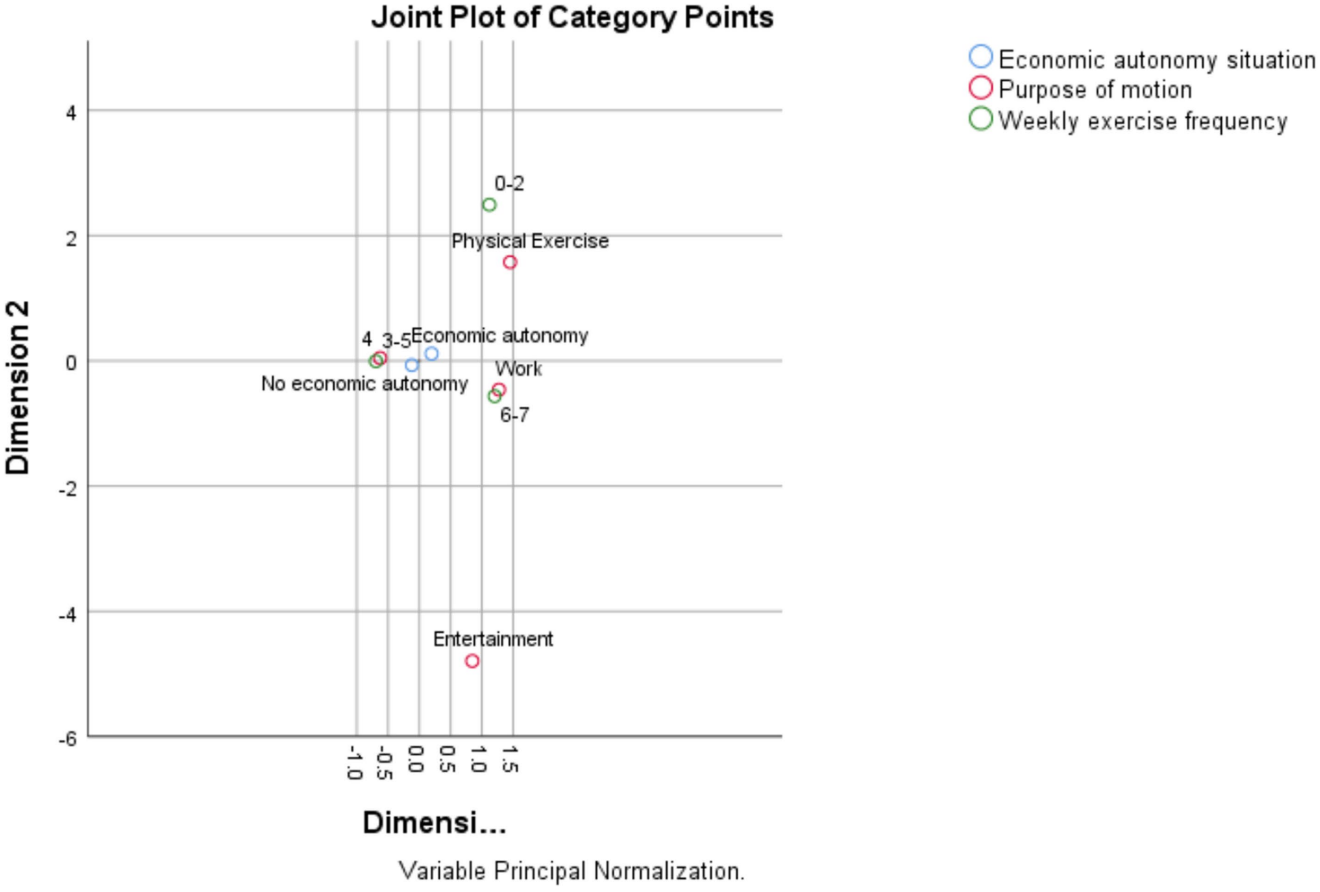
Moderate-intensity physical activity participation model.

**Figure 3:**
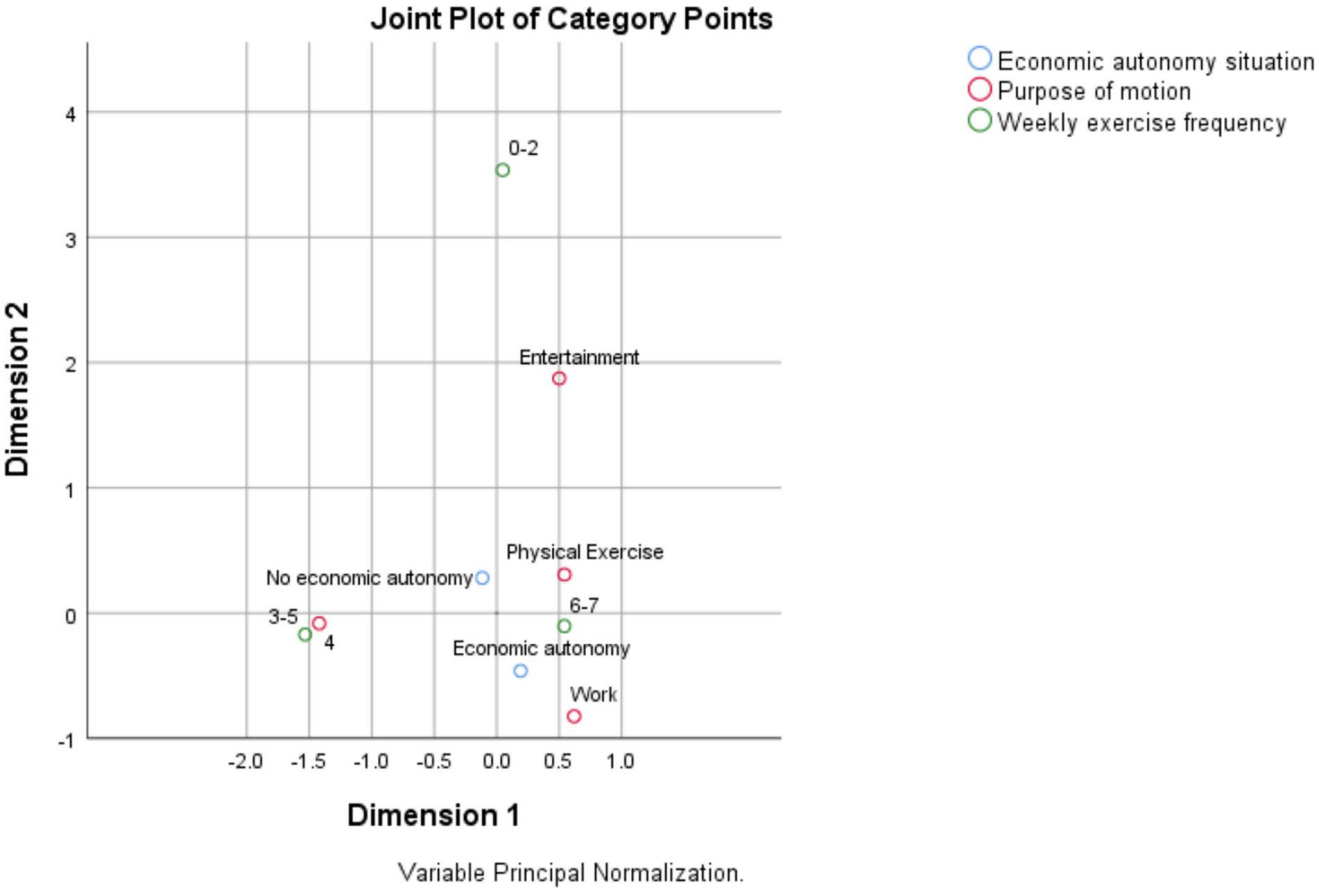
Low-intensity physical activity participation model.

As shown in the Figures 1–3, according to the physical activity characteristics of the older adult in China, the physical activity of the economically independent older adult group is defined as the active type, and the physical activity of the economically involuntary older adult group is defined as the passive type. The choice of physical activity of the active older adult group is less forced by external factors, and they can choose physical activity according to their own will. The choice of physical activity of passive active aged group is greatly affected by external factors, and their physical activity is mostly restricted by economic system. As shown in the figure, the economically independent older adult group in China has a higher initiative in the choice of physical activity, they can choose two types of physical activity, moderate intensity and low intensity. The economically independent group also has more diverse purposes of physical activity, and can have more choices in the medium intensity and low intensity physical activity. Economically independent older adult groups lack initiative in choosing physical activities. As shown, the choice of physical activities of these groups is highly related to work, and they can only engage in high-intensity physical activities and need to continue to engage in physical activities on a weekly basis, which is related to China’s work system.

## 4 Discussion

This study found that among Chinese older adult in both low and moderate-intensity physical activities, there is a significant correlation between economic autonomy and physical activity, which is consistent with existing research conclusions. However, in high-intensity physical activities, the economically non-autonomous group has a higher participation rate, contrary to existing research.

The uniqueness of physical activity among older adult people with different economic conditions in China alerts us to pay attention to the specificities of physical activity in different countries. A similar finding was discovered in a study conducted in Japan. During the research on Japanese adults, it was found that there are certain differences in the types of physical activities engaged in by individuals of different economic levels. High-income groups are more involved in physical activities related to entertainment and transportation, while adults with lower education levels have a higher participation in work-related physical activities (28). This suggests that attention should be paid to work-related physical activities in developing countries. Two other scholars’ studies have responded to our concern. In a study in Vietnam, it was found that the economically disadvantaged poor in Vietnam often have a higher participation in work-related physical activities (29). Similar findings were also made in studies of adults in Iran and Nepal (30, 31).

The physical activity patterns of economically independent older adults in China are characterized as active. These individuals can relatively freely select the type, timing, and frequency of physical activities that best suit their interests, health conditions, and available time. This autonomy not only contributes to improved physical health but also enhances their sense of social engagement and overall well-being. Conversely, the physical activity of economically dependent older adults in China is more constrained by external factors and is categorized as passive. Their participation in physical activities, including the type, frequency, and duration, is largely influenced by external systems. These systems limit their choices of physical activities, resulting in work-related physical activities that offer limited health benefit (32).

We understand the differences in physical activity types among older adult people with different economic conditions from both individual and environmental aspects. Firstly, China has a huge older adult population base, and the Chinese government faces significant financial pressure and is unable to provide a comprehensive social security system for the older adult. Secondly, the Chinese work system has limitations. Most Chinese enterprises and factories have not established a complete rest system. The phenomenon of overtime work such as “996” and “247” is severe, and the basic rights and interests of Chinese employees are not guaranteed (33). Many Chinese people do not have their own rest time, let alone engage in physical activities for exercise and entertainment. Thirdly, the economically non-autonomous group is limited by the level of education. The economically non-autonomous group often has a lower level of education, which limits their ability to obtain health information and choose appropriate physical activities, and they have insufficient awareness of self-active exercise (34, 35). They fail to correctly recognize the role of physical activity in physical and mental health and can only passively accept the influence of the environment. Again, the influence of the social support system is significant. A positive friend network system has a strong influence on the physical activity of the older adult (36). The economically autonomous and non-autonomous groups have different social support systems and living environments. Older adult people who are economically autonomous often have a higher level of education and self-awareness of exercise, which promotes the choice of physical activity among the older adult in the same network system. They actively choose suitable physical activity projects, jointly explore the benefits of physical activity, and jointly overcome the constraints such as insufficient facilities for physical activity among the current older adult (25). Finally, survival needs restrict physical activity. As of 2016, nearly 27% of the older adult in China had an income below the poverty line standard, and 43.5% of rural older adult had an income below the poverty line standard (37). They still need to work after the age of 60, and the division of labor in the economic system may also lead the economically non-autonomous group to engage only in high-intensity activities such as manual labor to meet their livelihood needs. These factors collectively contribute to the differences in economic autonomy in physical activities of different intensities. Future research can start from the constraints on physical activity of the economically non-autonomous group, such as how to solve the economic limitations of the economically non-autonomous older adult group, how to improve the insufficient awareness of physical activity among the economically non-autonomous older adult, or how to help the economically non-autonomous group establish a social support network aimed at physical activity, or help the economically non-autonomous older adult maintain a positive health status while balancing survival.

We have discovered different physical activity models for older adult people with different economic autonomy, which has significant implications for the formulation of sports public policies and the management of sports practices. Firstly, policymakers should pay attention to the differences in physical activity needs among older adult people with different economic autonomy. For the economically autonomous older adult group, policymakers should focus on how to better assist them in maintaining their physical activity habits and continuously stimulate their interest in physical activity. For the economically non-autonomous older adult group, policymakers need to provide sufficient economic support. The central government should increase investment in sports funds to enhance the economic autonomy of the older adult. Secondly, the Chinese government should further improve the work system. It is necessary to supervise labor-intensive enterprises in a timely manner to ensure they strictly adhere to the government-set working and rest hours, allowing employees to have their own genuine time. Thirdly, governments at all levels should jointly establish a multi-subject older adult sports service management system (38), encourage social forces to participate in older adult sports, and create a positive sports atmosphere throughout society to awaken the awareness of the economically non-autonomous older adult to actively engage in physical activities. Finally, grassroots healthcare providers should pay attention to the differences in physical activity among older adult people with different economic autonomy and grasp the key points of physical activity for the older adult. Many older adult people have chronic diseases and require correct medical intervention. It is necessary to promote the integration of sports and medical care and provide correct medical guidance to the current group of older adult people with chronic diseases. At the same time, grassroots managers should overcome the existing problems in sports social instructors, such as insufficient numbers of instructors, low guidance rates, and low guidance quality (39, 40), introduce more social instructors to participate in the guidance of physical activities for the older adult, and help the older adult establish correct sports concepts and exercise habits to actively participate in physical activities.

## 5 Conclusion

Economic autonomy is a determining factor affecting the participation of Chinese older adult in physical activity. The model shows that in moderate intensity and low intensity physical activity, the economy is positively correlated with physical activity, while the older adult with lower economic level engage in a lot of high intensity physical activity. Economically independent older adult people engaged in low-intensity physical activity mainly for work and exercise, high-intensity physical activity mainly for work; the reason why the older adult engaged in low-intensity physical activity was exercise, the main reason for participating in medium-intensity physical activity was other things and work, and the reason for participating in high-intensity physical activity was mainly for work. Our study breaks through the static cognition of the economic situation of the older adult and complements the relationship between economic conditions and physical activity of the older adult. The physical activity data of the older adult in China show that some of the older adult in the state of “economic non-autonomy” have more participation in high-intensity physical activity than the economically independent older adult, and need to spend longer time in high-intensity physical activity to maintain survival. This shows that after China has experienced rapid urbanization, many older adult people with low economic level are trapped in “survival activities” and do not have modern sports concepts. It is suggested that Chinese policy makers and practitioners should treat with caution the theoretical framework of the existing research that the better the economic level, the higher the physical activity, and avoid increasing the excessive physical activity of the older adult in the lower economic level. Policy makers should base on the special economic and physical activities of the older adult in China, promote the adaptation of the older adult to modern sports concepts, maintain the physical fitness and physical activity ability of the older adult through appropriate types of physical activities (such as exercise prescription), and improve the physical health and living standards of the older adult. At the same time, we should pay attention to support the older adult from the economic level and improve the degree of economic autonomy of the older adult. Due to the limitation of time and ability, we only studied the relationship between economic autonomy and physical activity. How to help the older adult with different types of economic autonomy to carry out targeted physical activity and improve the quality of life of the older adult needs to be further explored. Future studies can be further discussed on this basis. It should be noted that the economic situation of the older adult in China is influenced by China’s special historical development stage and culture. The proportion of the existing older adult who have received higher education is low, which makes it difficult for them to adapt to the social division of labor in the current economic system. Meanwhile, China’s culture of filial piety and human network will have an impact on the economic situation of the older adult. When expanding to other areas, it is necessary to consider the specific local conditions and make policy adjustments according to the specific circumstances.

## Data Availability

The datasets presented in this study can be found in online repositories. The names of the repository/repositories and accession number(s) can be found at: https://charls.charlsdata.com/pages/Data/2018-charls-wave4/zh-cn.html.
